# Brief report: the association between recreational versus therapeutic marijuana use on hazardous alcohol consumption and alcohol-associated behavioral consequences among adults living with HIV in Florida

**DOI:** 10.1186/s12954-018-0266-2

**Published:** 2018-12-07

**Authors:** Zachary L. Mannes, Erin G. Ferguson, Larry E. Burrell, Robert L. Cook, Nicole Ennis

**Affiliations:** 1 Department of Clinical and Health Psychology, College of Public Health and Health Professions, University of Florida, P.O. Box 100165, 1225 Center Drive, Room 3146, Gainesville, FL 32610-0165 USA; 2 Psychology Service, San Francisco VA Medical Center, 4150 Clement St., San Francisco, CA 94121 USA; 3 Department of Epidemiology, College of Public Health and Health Professions and College of Medicine, University of Florida, P.O. Box 100231, 2004 Mowry Road, Gainesville, FL 32610 USA

**Keywords:** HIV/AIDS, Hazardous alcohol use, Marijuana use

## Abstract

**Background:**

Though marijuana use has previously been associated with risky alcohol use, studies often do not delineate between the effect of recreational versus therapeutic marijuana use, particularly among people living with HIV (PLWH). In this study, we examined the association between recreational versus therapeutic marijuana use to manage HIV symptoms (i.e., improve appetite/gain weight, induce sleep, relieve nausea/vomiting, relieve pain, relieve anxiety/depression/stress) on hazardous alcohol consumption and associated behavioral consequences among PLWH.

**Methods:**

PLWH (*N* = 703) recruited from community health centers in Florida completed questionnaires assessing sociodemographics, marijuana use motives (i.e., recreational versus therapeutic), alcohol use, and alcohol-associated behavioral consequences. Hazardous alcohol use was defined as consuming 5 or more drinks on one occasion at least monthly or > 14 drinks per week for men, or 4 drinks on one occasion at least monthly or > 7 drinks per week for women over the past 12 months, while alcohol-associated behavioral consequences were assessed via the Short Inventory of Problems Revised (SIP-R). A one-way analysis of covariance (ANCOVA) assessed differences in average number of alcohol-associated behavioral consequences between recreational and therapeutic marijuana users, and non-users, while multivariate logistic regression analysis evaluated the association between reason for marijuana use and hazardous alcohol consumption.

**Results:**

There was a significant effect of marijuana use group on SIP-R score after controlling for covariates [*F* (2, 579) = 3.04, *p* = 0.048], with post hoc analysis demonstrated significantly fewer alcohol-associated behavioral consequences among therapeutic marijuana users (1.27) compared to recreational users (3.35; *p* = 0.042). Compared to non-users, therapeutic marijuana users demonstrated significantly lower odds of hazardous drinking (AOR = 0.42, 95% CI = 0.18–0.96, *p* = 0.041), while recreational marijuana users were 64% more likely to report hazardous drinking (AOR = 1.64, 95% CI = 1.08–2.50, *p* = 0.019).

**Conclusions:**

Findings from this study add to the literature by demonstrating how differing marijuana use motives are associated with hazardous alcohol consumption among PLWH. Given our findings showing greater risk of hazardous alcohol consumption among recreational marijuana users and lower risk among therapeutic marijuana users, results from this study may help inform interventions to reduce harmful alcohol consumption and associated adverse consequences among PLWH.

## Background

Hazardous alcohol use is defined by having 5 or more drinks on one occasion (at least monthly) or > 14 drinks per week for men, and 4 drinks on one occasion (at least monthly) or > 7 drinks per week in the past 12 months for women [1–3]. Previous studies have found that hazardous alcohol use is more prevalent among people living with HIV (PLWH) compared to the general population and is associated with missed clinic appointments, decreased retention in care, suboptimal antiretroviral adherence, and increased morbidity and mortality [4–8].

Though factors associated with hazardous alcohol consumption include depression, illicit drug use, and marijuana use, hazardous alcohol use and alcohol-associated behavioral consequences may differ based on whether PLWH use marijuana recreationally or for therapeutic purposes to manage HIV-associated medical symptoms [9]. In the USA, up to 56% of PLWH report current marijuana use [10]. While many PLWH report recreational motives for use, approximately 60% report use to manage HIV-associated symptoms such as pain, nausea, lack of appetite, insomnia, and depression [10–12].

The literature examining the effect of medical versus recreational marijuana use on alcohol consumption behaviors remains inconclusive [13]. Though medical marijuana is often considered that prescribed by a physician, marijuana is one of the most common types of complementary and alternative medicines used by adults living with HIV in Florida, with many PLWH reporting self-management of medical symptoms as the primary reason for use [14]. Given that motives of alcohol use among PLWH include managing HIV-associated medical symptoms such as pain, stress, and depression, treatments (i.e., marijuana use) to alleviate similar symptoms may influence alcohol consumption patterns in this population, as previous studies have shown that alcohol drinkers reduce use when marijuana is an accessible alternative to relieve medical symptoms [15, 16]. To this point, results from a recent study demonstrated a significantly lower prevalence of hazardous alcohol use among therapeutic marijuana users (i.e., persons using marijuana to self-manage specific symptoms or health conditions; 24.0%) compared to recreational marijuana users (49.1%) and non-users (32.4%) [9]. Building upon these results, in this study, we examined the effect of recreational versus therapeutic marijuana use on hazardous alcohol consumption and alcohol-associated behavioral consequences among PLWH in Florida. We hypothesized that compared to non-users, therapeutic marijuana users would demonstrate significantly lower odds of hazardous drinking while recreational marijuana users would be more likely to report hazardous drinking. We further hypothesized that therapeutic marijuana users would report significantly fewer alcohol-associated behavioral consequences compared to recreational marijuana users and non-users.

## Methods

### Participants and procedures

Study methods have been previously detailed [17]. Briefly, the sample (*N* = 703) included participants recruited from 2014 to 2017 for the Florida Cohort Study, an investigation examining determinants of health outcomes of PLWH in Florida. The Florida Cohort Study utilizes a convenience-sampling method of data collection across multiple county health departments and community setting clinics throughout Florida (Gainesville, Ft. Lauderdale, Lake City, Miami, Orlando, Sanford, and Tampa). All PLWH 18 and older at the selected recruitment settings were eligible to participate. After providing written informed consent, participants completed questionnaires assessing sociodemographics, depressive symptoms, and substance use. Participants received $25.00 after study completion. The Institutional Review Boards of the University of Florida, Florida International University, and Florida Department of Health approved this study.

### Measures

#### Marijuana use

Participants were asked if they used any marijuana in the past 3 months. Since data collection for this study occurred prior to the 2017 legalization of physician-prescribed medical marijuana in Florida, recreational and therapeutic marijuana users were defined based on self-reported motives for use, in line with prior research in collaboration with the CDC [18]. Recreational marijuana users endorsed use to get high or stoned, increase libido/improve sexual performance, or fit into social situations. Therapeutic marijuana users reported use to improve appetite/gain weight, induce sleep, relieve nausea/vomiting, relieve pain, and relieve anxiety/depression/stress. Participants endorsing both recreational and therapeutic marijuana use were classified as recreational users, whereas therapeutic users were those reporting therapeutic use only.

#### Hazardous alcohol use

Participants reported the average frequency and quantity of consumption of standard alcoholic beverages in the past year. Average weekly consumption was calculated by multiplying the average daily quantity by the average frequency per week. Participants also reported on how often they drank 4+ standard drinks (for women) or 5+ standard drinks (for men) on one occasion, as well as the largest number of drinks they consumed within a 24-h period. Participants were classified as either hazardous or non-hazardous users based on their report of meeting hazardous drinking criteria (i.e., 5 or more drinks on one occasion at least monthly or > 14 drinks per week for men, or 4 drinks on one occasion at least monthly or > 7 drinks per week for women) [1–3].

#### Alcohol-associated behavioral consequences

The Short Inventory of Problems Revised (SIP-R) is a valid and reliable questionnaire for measuring alcohol-associated negative consequences related to physical or monetary harm, relationship discord, and impulsive decision-making over the past 30 days. Participant SIP-R scores ranged from 0 to 15, and the scores were averaged and compared among recreational and therapeutic marijuana users and non-users [19].

#### Durable viral suppression

HIV viral suppression was defined as all viral load tests suppressed (≤ 200 copies/ml) in the past year, based on information available from statewide HIV surveillance data.

#### Other substance use

Participants were dichotomized as to whether they reported use of any illicit drugs other than marijuana (i.e., ecstasy, crack/cocaine, nonprescription opioid use, and injection drugs) in the past year.

#### Depressive symptoms

The Patient Health Questionnaire (PHQ-8), a reliable and valid instrument for PLWH, was used to define current depressive symptoms (i.e., a score of ≥ 10), consistent with prior research [20, 21].

#### Sociodemographic factors

Age, race, sex, education, sexual identity, years since diagnosis, and homelessness were assessed. For the purpose of our analyses, participants were categorized into four age groups: 18–34, 35–44, 45–54, and ≥ 55. Race/ethnicity was categorized into Hispanic, White, Black, and Others. Sex was based on participant’s assigned sex at birth (i.e., male or female). Level of education was classified into three groups: less than high school, high school, and more than high school. Sexual identity was categorized as heterosexual, homosexual, or bisexual. Years since diagnosis was a continuous variable assessing number of years since HIV diagnosis. Homelessness was defined as living in a homeless shelter, emergency shelter, car, street, or abandoned building in the last year.

### Statistical analyses

Analyses were conducted in SPSS version 24 [22]. Descriptive statistics were used to analyze sociodemographic factors and the prevalence of depressive symptoms, illicit drug use, motives of marijuana use, and hazardous drinking. Sex, age, race, education, sexual identity, homelessness, depressive symptoms, other drug use, years since diagnosis, and durable viral suppression were included as covariates in multivariate analysis, as prior research suggests associations with risky alcohol use among PLWH [5, 7, 8]. A one-way analysis of covariance (ANCOVA) with adjustment for the aforementioned covariates assessed differences in average number of alcohol-associated behavioral consequences between recreational users, therapeutic marijuana users, and non-users. Following observation of a significant main effect, pairwise comparisons between the three groups were made using the Bonferroni correction. The level of significance was set at *p* < 0.05. Multivariate logistic regression assessed the association between therapeutic versus recreational marijuana use and hazardous drinking. Adjusted odds ratios with 95% confidence limits were presented with non-users as the referent group.

## Results

### Sample characteristics

The sample (*N* = 703) had a mean age of 47 years (SD = 11.27). The racial/ethnic breakdown was 53.7% Black, 22.4% White, 20.4% Hispanic, and 3.5% Other. Most participants (68.7%) were males, and 62.2% reported completing high school or less. The majority (81.0%) reported being unmarried. Mean years since HIV diagnosis was 11.52 (SD = 7.61). Approximately 36% of PLWH reported hazardous alcohol consumption (Table 1).

**Table 1:** Demographics, substance use, mental health, and health status (*N* = 703)

Variable	Category	Total (*N* = 703)
Demographics		
Age	18–34	112 (16.2)
	35–44	124 (17.9)
	45–54	279 (40.4)
	≥ 55	176 (25.5)
Sex	Male	475 (68.7)
	Female	216 (31.3)
Race	Hispanic	141 (20.4)
	Not Hispanic, White	155 (22.4)
	Not Hispanic, Black	371 (53.7)
	Not Hispanic, Other	24 (3.5)
Education	< High school	220 (31.9)
	High school diploma or equivalent	208 (30.2)
	> High school	261 (37.9)
Sexual identity	Heterosexual	341 (51.2)
	Homosexual	260 (39.0)
	Bisexual	65 (9.8)
Homelessness	No	576 (84.5)
	Yes	106 (15.5)
Substance use		
Marijuana use	No	469 (66.8)
	Yes	234 (33.2)
Other drug use	No	416 (63.2)
	Yes	242 (36.8)
Hazardous drinking	No	417 (63.9)
	Yes	236 (36.1)
Mental health		
Depressive symptoms	No	465 (68.2)
	Yes	216 (31.8)
Health status		
Viral suppression	No, at least once > 200	263 (40.2)
	Yes, all ≤ 200	390 (59.8)

Note: *N* may vary slightly according to missing data

### Recreational and therapeutic marijuana use

**Fig. 1:**
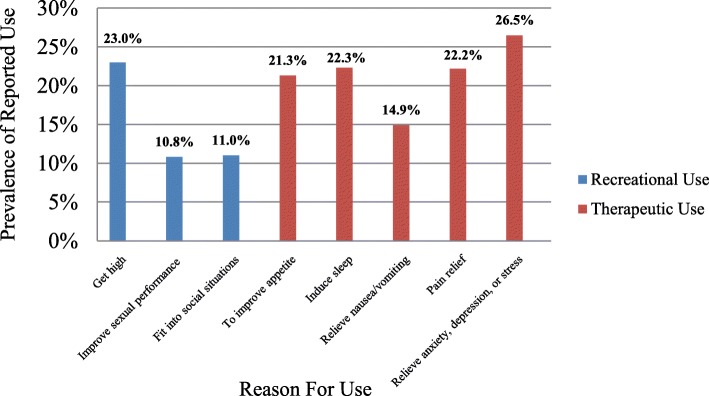
Prevalence of reported recreational and therapeutic reasons for marijuana use (*n* = 246). Note: Participants could select more than one option

Marijuana use in the past 3 months was reported by 33.2% of participants. After categorization, 51 (7.2%) of all participants were therapeutic users, 183 (26.0%) were recreational users, and 469 (66.8%) were non-users (Fig. 1). Additional information related to participant marijuana use and motives of use have been published elsewhere [9].

### The association between marijuana use motive, alcohol-associated consequences, and hazardous drinking

Therapeutic marijuana users reported 1.27 alcohol-associated behavioral consequences compared to 3.35 and 2.79 for recreational users and non-users respectively. After controlling for sex, age, race, education, sexual identity, homelessness, depressive symptoms, other drug use, years since diagnosis, and durable viral suppression, there was a significant effect of marijuana use motive on the total number of reported drinking consequences [*F* (2, 579) = 3.04, *p* = 0.048]. Bonferroni-corrected post hoc analysis demonstrated a significant difference in SIP-R score between therapeutic marijuana users and recreational users (*p* = 0.042), while no significant difference was observed between therapeutic users and non-users (*p* = 0.100), nor recreational users and non-users (*p* = 0.374). Compared to non-users, therapeutic marijuana users had significantly lower odds of reporting hazardous drinking (AOR = 0.42, 95% CI = 0.18–0.96, *p* = 0.041) while recreational marijuana users had approximately a 64% greater odds of hazardous drinking (AOR = 1.64, 95% CI = 1.08–2.50, *p* = 0.019) after controlling for the aforementioned covariates (see Table 2 and Table 3).

**Table 2:** ANCOVA examining the association between recreational and therapeutic marijuana use and alcohol-associated behavioral consequences (*N* = 703)

Variable	*F*	*p* value	*η* ^2^	*M*, non-user	*M*, therapeutic user	*M*, recreational user
Marijuana	*3.04*	*0.048*	*.011*	*2.79*	*1.27*	*3.35*
Covariates						
Sex	2.70	0.311	.002			
Age	0.31	0.860	.000			
Race	2.54	0.111	.004			
Education	*4.33*	*0.038*	*.008*			
Sexual identify	0.73	0.392	.001			
Homelessness	*14.56*	*< 0.001*	*.025*			
Depressive symptoms	1.96	0.162	.003			
Other drug use	*26.05*	*< 0.001*	*.044*			
Years since diagnosis	1.38	0.239	.002			
Durable viral suppression	0.00	0.960	.000			

Note: *M* mean number of alcohol-associated behavioral consequences. Italicized values indicate significance at *p*<0.05. Adjusted for sex, age, race, education, sexual identity, homelessness, depressive symptoms, other drug use, years since diagnosis, and durable viral suppression

**Table 3:** Multivariate logistic regression analysis examining the association between marijuana use motive and hazardous alcohol use (*N* = 703)

Variables	AOR (CI)	*p* value
Marijuana use		
No marijuana use	Referent	
Therapeutic use	*0.42 (0.18–0.96)*	*0.041*
Recreational use	*1.64 (1.08–2.50)*	*0.019*
Sex		
Male	Referent	
Female	0.92 (0.56–1.50)	0.739
Age		
18–34	Referent	
35–44	0.96 (0.51–1.81)	0.919
45–54	0.81 (0.44–1.47)	0.491
≥ 55	0.95 (0.47–1.92)	0.906
Race		
Not Hispanic, White	Referent	
Not Hispanic, Black	1.62 (0.91–2.88)	0.098
Not Hispanic, Other	1.15 (0.68–1.95)	0.587
Hispanic	1.32 (0.46–3.78)	0.602
Education		
< High school	Referent	
High school	0.83 (0.52–1.33)	0.459
> High school	*0.61 (0.38–0.99)*	*0.048*
Sexual identity		
Heterosexual	Referent	
Homosexual	1.04 (0.63–1.72)	0.866
Bisexual	0.71 (0.35–1.41)	0.332
Homelessness		
No	Referent	
Yes	1.47 (0.89–2.43)	0.124
Depressive symptoms		
No	Referent	
Yes	1.29 (0.86–1.92)	0.204
Other drug use		
No	Referent	
Yes	*2.30 (1.57–3.37)*	*< 0.001*
Years since diagnosis		
	1.01 (0.98–1.04)	0.234
Durable viral suppression		
No	Referent	
Yes	*0.67 (0.45–0.99)*	*0.047*

Note: Italicized values indicate significance at *p*<0.05. Adjusted for sex, age, race, education, sexual identity, homelessness, depressive symptoms, other drug use, years since diagnosis, and durable viral suppression

## Discussion

The purpose of this study was to examine the association between marijuana use motives, alcohol-associated behavioral consequences, and hazardous alcohol consumption in a sample of PLWH in Florida. Results demonstrated that therapeutic marijuana users had significantly lower odds of hazardous drinking compared to non-users and also reported significantly fewer alcohol-associated behavioral consequences compared to recreational users. Conversely, recreational users demonstrated greater odds of hazardous drinking compared to non-users. These results suggest that therapeutic marijuana use, rather than recreational marijuana use, may be most useful for minimizing the harmful effects of hazardous alcohol use and associated behavioral consequences. Given these findings, clinicians may be able to evaluate patient risk for hazardous drinking by assessing their rationale for marijuana use.

Complementing findings from the general population, our results indicate that recreational marijuana use confers the greatest risk for hazardous alcohol use and alcohol-associated consequences [23, 24]. This may be a function of the different motives for use. Unlike PLWH reporting therapeutic marijuana use to alleviate health symptoms, recreational users had varying motives, including getting high/stoned, fitting into social situations, and improving sexual performance. In particular, using marijuana to get high/stoned and to fit into social situations may be implicated in recreational users’ alcohol consumption, as these social situations could also facilitate alcohol use [25]. Moreover, evidence suggests that recreational users are more likely to report higher impulsivity, sensation seeking, and disinhibition than non-users in the general population [26]. Therefore, it is possible that recreational users in this sample used alcohol to seek stimulating experiences associated with potential negative consequences.

Compared to recreational users and non-users, therapeutic marijuana users demonstrated a lower prevalence of hazardous drinking and fewer reported behavioral consequences as a result of their alcohol consumption. Given this, therapeutic users in this study may have substituted their alcohol consumption for marijuana use due to the known deleterious effects of risky alcohol use, and because they perceived marijuana as a more effective means to manage HIV-associated symptomology. Previous studies have shown that individuals decreased their use of alcohol in locales where medical marijuana was legalized [16, 27, 28]. Lucas and colleagues reported that 41% of participants used marijuana as a substitute for alcohol, with 60% noting making this substitution as a result of experiencing fewer side effects, and 52.9% reporting substitution as a result of more effective medical symptom management with marijuana [16]. Therapeutic users in this study may have engaged in marijuana substitution for similar reasons, resulting in reduced alcohol consumption and associated consequences. Existing literature also suggests that medical marijuana users in the general population are more likely to endorse daily use and have significantly more medical and psychological comorbidities than recreational marijuana users [29]. Therefore, therapeutic users in this sample may have been more likely to use marijuana for treatment purposes and thus limit their use of alcohol due to perceived negative health consequences [30].

Our investigation examining differences in alcohol consumption behaviors yielded novel findings and has some limitations. First, our study utilized a cross-sectional design; thus, we cannot determine the temporality of our findings. Additionally, all participants were PLWH enrolled in care within Florida and thus our findings may not generalize to all PLWH using marijuana. It is also possible that recreational marijuana users in this study may have been engaging in simultaneous substance use (i.e., using marijuana and alcohol at the same time), which was not assessed by the Florida Cohort Study. Despite these limitations, this study found salient differences in alcohol use behaviors between recreational and therapeutic marijuana users. To the author’s knowledge, this is the first study examining the association between recreational versus therapeutic marijuana use while controlling for significant covariates in a sample of PLWH.

## Conclusions

Results from this study add to the literature by demonstrating how differing rationales of marijuana use are associated with hazardous alcohol consumption among PLWH. Findings demonstrating greater risk of hazardous alcohol consumption among recreational marijuana users and lower risk among therapeutic marijuana users may help inform interventions to reduce harmful alcohol consumption and prevent adverse consequences among PLWH. Continued research examining the effect of different motives for marijuana use, specifically physician-prescribed marijuana use, on alcohol consumption behaviors among PLWH is important as marijuana legalization proliferates.

## Abbreviations

ANCOVA: One-way analysis of covariance
CDC: Center for Disease Control and Prevention
PLWH: People living with HIV
